# Spontaneous Bilateral Tubal Gestation: A Rare Case Report

**DOI:** 10.1155/2016/8526903

**Published:** 2016-07-17

**Authors:** Marwah Sheeba, Gupta Supriya

**Affiliations:** ^1^Department of Obstetrics & Gynecology, VMMC and Safdarjung Hospital, New Delhi 110029, India; ^2^Department of Obstetrics & Gynecology, Government Multi-Specialty Hospital, Chandigarh 110016, India

## Abstract

Here a case is presented where the woman after a positive pregnancy test underwent medical abortion for unwanted pregnancy without ultrasound confirmation of intrauterine pregnancy. On persistence of symptoms, a second opinion was procured, when examination and a transvaginal ultrasound scan revealed ruptured unilateral tubal ectopic pregnancy. However upon subsequent laparotomy (considering deteriorating hemodynamic status of the patient), intraoperatively it turned out to be a bilateral tubal ectopic gestation.

## 1. Introduction

The rarity with which one comes in contact with bilateral tubal pregnancy in the literature accentuates the infrequency of this malady. Incidence is about 1 in 725 to 1 in 1580 ectopic pregnancies and 1 in 2 lac intrauterine pregnancies [1–5]. Around 200 cases of bilateral tubal ectopic pregnancy have been reported in the literature till date [6]. However a significant escalation in its incidence has been noted over the last three decades [7]. It usually ensues ovulation stimulation as in artificial reproductive techniques, pelvic infection, and tubal surgeries [8, 9]. There are not many cases reported with preoperative diagnosis of bilateral ectopic pregnancy [1, 10]. The case described here also was diagnosed intraoperatively, but occurred spontaneously [1, 11, 12].

## 2. Case

A 28-year-old lady, gravida 3 para 2 live 2 with previous one LSCS, married for 8 years arrived in gynecological emergency of the institute with two months of amenorrhea, excruciating pain abdomen ×1 day, and 5-6 episodes of vomiting ×1 day. She had not been using any contraception. Her previous menstrual cycles were regular of thirty days with bleeding period of 3 days. Upon missing her periods and a positive UPT at home, she took MTP pill as advised by a local doctor for unwanted pregnancy. However in event of RPOCs, she underwent a dilatation and curettage at a private hospital in the vicinity of her residence. Following the same, pain abdomen and spotting per vaginum still persisted. When abdominal pain became unbearable she was brought to hospital. Her past medical and family history were not significant.

Her general condition on admission was guarded with maternal tachycardia (pulse 110 per minute), hypotension (BP = 70/40 mmHg), RR = 20/min, and pallor. Her abdomen was tender on palpation with positive rebound tenderness and guarding and absent bowel sounds. Pelvic bimanual examination revealed a bulky uterus with cervical excitation and bilateral adnexal fullness and tenderness that was more marked in the right fornix. A transvaginal ultrasound scan showed an empty uterus with thickened endometrium and a right adnexal mass that was suggestive of a right tubal pregnancy with a moderate amount of free fluid in the pouch of Douglas. Culdocentesis done was positive. Her investigations revealed Hb 7.6 gm%, TLC 12,200, DLC N88 L12, platelets 3.46 Lac, PT, PTTK normal, blood urea 24, creatinine 0.4, Na 131, and K 3.9. Ultrasound revealed ruptured left tubal pregnancy with significant free fluid in the abdomen and pelvis. There was no obvious ultrasonic evidence of right tubal disease.

**Figure 1:**
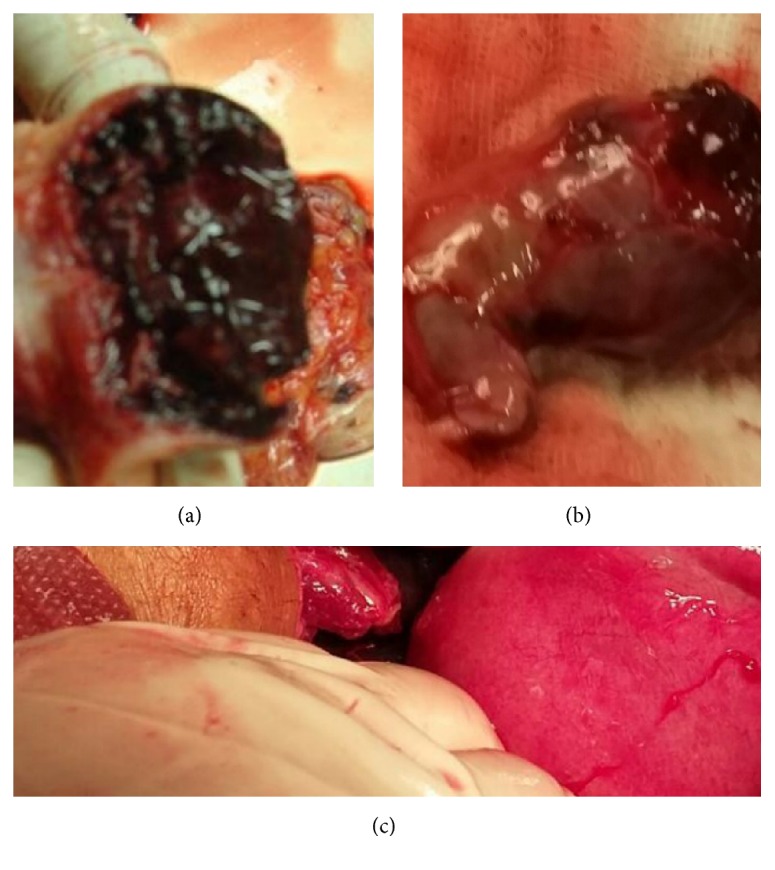
Gross picture showing organized hematoma on left side (a), ruptured right tubal ectopic pregnancy (b), and normal uterus (c).

**Figure 2:**
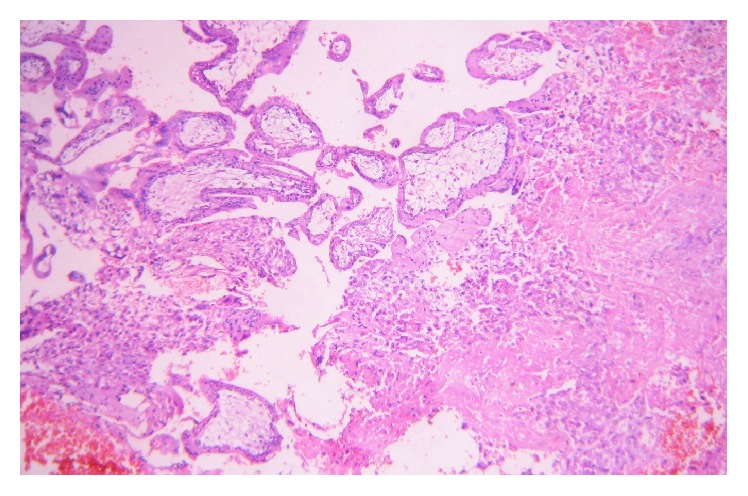
Case-tube one histopathology.

**Figure 3:**
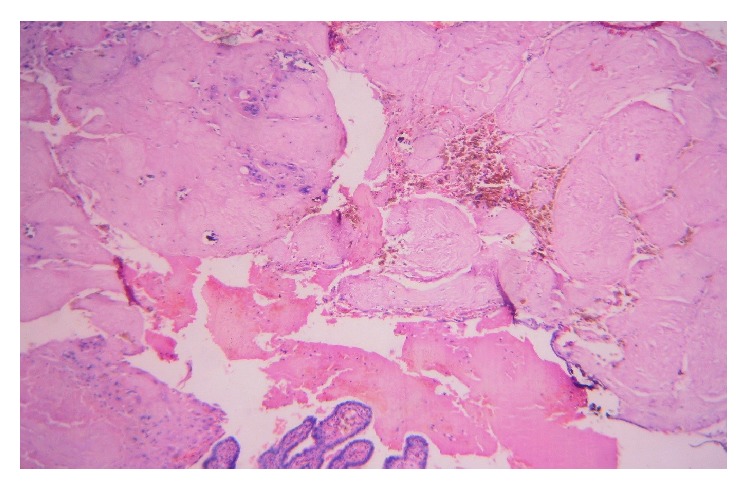
Case-tube two histopathology.

She was resuscitated and taken up in emergency for exploratory laparotomy and proceed in view of deteriorating vitals, after written informed consent. Consent for tubal ligation was also taken because she was multiparous. Preoperatively 2 litres of hemoperitoneum was drained. There was a ruptured left sided ampullary ectopic pregnancy ~6 × 3 cm; right tube revealed *⋍*3 × 3 cm organized hematoma seen attached to fimbrial end. Uterus and bilateral ovaries are normal (Figure 1). A bilateral salpingectomy was hence performed. She was transfused three units of packed red blood cells intraoperatively. She had an unremarkable postoperative recovery and was discharged on the third postoperative day. Histopathological examination showing chorionic villi and trophoblasts in both tubes confirmed the diagnosis of bilateral tubal ectopicpregnancy with evidence of rupture on one side (Figures 2 and 3).

## 3. Discussion

Tubal ectopic pregnancy is a familiar, yet life-threatening entity encountered by obstetricians, necessitating prompt and precise management. 2% of all first trimester pregnancies are ectopic pregnancies [9]. Mortality rates for ectopic pregnancy are high, being the leading cause of maternal death in first trimester, accounting for 9–13% of all pregnancy related deaths [9, 13, 14]. Bilateral tubal ectopic pregnancy is the rarest form of extra-uterine pregnancy and is usually associated with infertility treatment. Simultaneous rupture of tubes is even rarer. Twin tubal pregnancy with both embryos in the same tube as well as with one in each tube has also been stated [15, 16].

Multiple ovulation fosters transperitoneal migration of trophoblastic cells along with superfetation. Also second tubal pregnancy following demise of the first one has also been postulated to be a cause of bilateral ectopic. Besides, increased incidence of the same could be attributed to increased rate of sexually transmitted infections that damage the fallopian tubes, the use of antibiotic treatments for pelvic inflammatory disease, increased use of assisted reproductive technologies, and increased rates of tubal sterilization, smoking, and polygamy, besides more accurate methods for early detection of ectopic pregnancy. Prior tubal damage confers the highest risk for ectopic pregnancy [17]. The uniqueness of our case is further augmented by the conspicuous absence of the aforesaid usual risk factors in the woman. However in such a case also, the clinical suspicion must still remain high to detect the ectopic gestation especially bilateral one [9, 18, 19].

Complications of ectopic pregnancy are resultant of misdiagnosis, delayed diagnosis, or faulty management approach. Inability to clinch a prompt and accurate diagnosis of ectopic pregnancy can cause tubal or uterine rupture (depending on the location of the pregnancy), which in turn can lead to massive hemorrhage, shock, disseminated intravascular coagulopathy (DIC), and/or death [9].

Presenting features are constituted by the usual triumvirate of amenorrhea, vaginal bleeding, and abdominal pain. However diagnosis utilizing levels of *β*-HCG discriminatory zone is not reliable in case of bilateral ectopic gestation. Meticulous preoperative and intraoperative evaluations are needed to evade inapposite treatment, such as removal of the contralateral tube. Ultrasonography is a nifty investigative tool to elucidate the diagnosis; however every now and then it can be deceptive [20, 21]. Color Doppler capacities further enhance the diagnostic sensitivity of transvaginal ultrasound for the early recognition of abnormal and normal intrauterine pregnancy and small extra-uterine masses preoperatively. Literature review has proven it to be an operative diagnosis like the case in question, barring only few reports [22]. Frequently, there is a failure to recognize the entity during surgery as well. In such cases, various criteria validated in the past become useful for opinion involving either description of fetal parts as well as of placental material or microscopic identification of chorionic villi in each tube [10, 23].

Currently the principal management in ectopic pregnancy has become conservative with the aim of salvaging the tube, rather than salpingectomy. Despite the risk of persistent ectopic pregnancy, some studies have shown salpingostomy to have improved reproductive outcome in patients with contralateral tubal damage [9]. In the featured case, left salpingectomy was performed because of uncontrolled bleeding from the implantation site with severely damaged tube and large tubal pregnancy. Also since a prior consent for ligation was already there, no second thoughts crept into the minds after visualizing the right side findings of an organized hematoma.

Comparing salpingectomy by laparotomy or laparoscopy, former is preferred when patient is hemodynamically unstable like the case in discussion; though subsequent intrauterine pregnancy rates are higher in laparotomy. The rate of persistent ectopic pregnancy between the two approaches is similar. However laparoscopic approach is feasible subject to availability of expertise and infrastructure at the attending hospital [24, 25].

One must exercise vigilance postoperatively by conducting follow-up tests like serial measurement of serum concentrations of human chorionic gonadotrophin to exclude the risk of persistent trophoblast in cases of conservative surgery [26, 27]. In our case since salpingectomy was opted for, it was not warranted.

## 4. Conclusion

Even in this era of highly advanced medical technology, simple dictum of “think ectopic” must not be forgotten, especially in conception following artificial reproductive techniques. Besides, a high index of suspicion and diligent examination of the adnexa at the time of surgery is warranted to identify bilateral ectopic gestation.
